# Differences in the structure of outpatient diabetes care between endocrinologist- led and general physician- led services

**DOI:** 10.1186/1472-6963-13-493

**Published:** 2013-11-25

**Authors:** Máire O’Donnell, Anna de Siún, Monica O’Mullane, Diarmuid Smith, Colin Bradley, Francis M Finucane, Sean F Dinneen

**Affiliations:** 1 Clinical Science Institute, Galway Diabetes Research Centre and HRB Clinical Research Facility, School of Medicine, National University Ireland, University Road, Galway, Ireland; 2 Irish College of General Practitioners, Dublin, Ireland; 3 Department of General Practice, University College Cork, Cork, Ireland; 4 Department of Endocrinology, Beaumont Hospital, Dublin, Ireland

**Keywords:** Structured diabetes care, Hospital outpatients, Endocrinologist, General physician

## Abstract

**Background:**

Despite a shift in diabetes care internationally from secondary to primary care, diabetes care in the Republic of Ireland remains very hospital-based. Significant variation in the facilities and resources available to hospitals providing outpatient diabetes care have been reported in the UK. The aim of this study was to ascertain the structure of outpatient diabetes care in public hospitals in the Republic of Ireland and whether differences existed in services provided across hospitals.

**Methods:**

We conducted a cross sectional observational study of the 36 public general hospitals providing adult outpatient diabetes care in the Republic of Ireland. Data relating to numbers of specialist medical, nursing and allied health professionals, waiting times for new and return patients, patterns of discharge back to primary care and engagement in quality improvement initiatives were recorded.

**Results:**

Thirty-five of the 36 eligible hospitals participated in the study. Sixty percent of these had at least one consultant endocrinologist in post, otherwise care delivery was led by a general physician. Waiting times for newly diagnosed patients exceeded six months in 30% of hospitals and this was higher where an endocrinologist was in place (47% V 7%, p = 0.013). Endocrinologists were more likely to have developed subspecialty clinics, access to allied health professionals and engage more in quality improvement initiatives but less likely to discharge patients back to primary care than general physicians (76 v 29%, p = 0.005).

**Conclusions:**

Variations exist in the structure and provision of diabetes care in Irish hospitals. Endocrinology-led services have more developed subspecialty structures and access to specialist allied health professionals and engage more in quality improvement initiatives. Nonetheless, waiting times are longer and discharge rates to primary care are lower than for non-specialty led services. Further studies to determine the extent to which case-mix variation accounts for these observations are warranted. Aspects of hospital-based outpatient care could be developed further to ensure equitable services are provided nationally. At a time when the delivery of diabetes services in primary care is being promoted, further research is warranted on the factors influencing the successful transition to primary care.

## Background

The rising prevalence of diabetes mellitus in the context of limited health care resources poses a major clinical and health economic challenge for optimal delivery of care to affected individuals. As the emphasis moves towards secondary prevention of diabetes complications, there has been a shift from hospital- to community-based care. This has been shown to be effective where adequate structures and supports are in place [1-3].

Recent Irish policy documents have promoted “integrated care” where the routine management of uncomplicated type 2 diabetes will shift to primary care while those with complicated type 2 diabetes will be managed between primary and secondary care [4,5]. Although specific structured or shared care programmes for diabetes have been developed in some areas [2,6], recent research suggests that the delivery of diabetes care in primary care remains largely unstructured [7] and in many areas the delivery of diabetes care remains very hospital-based.

A number of factors contribute to the current delivery of diabetes care in Ireland. The Irish health care system is a mix of public and private health service funding with about a third of the population holding medical cards that entitle them to free services including primary care [8]. The qualification criteria for these medical cards are largely income-related. The remaining population is not entitled to free primary health care. For diabetes patients this means all patients have access to free public outpatient diabetes care but non-medical card holders usually have to pay for diabetes services delivered by their GPs. Also, unlike their counterparts in the UK, Irish GPs are currently not reimbursed for delivering diabetes care. Support services such as podiatry, dietetics, retinal screening and specialist nursing staff in the community have not yet been optimally established.

Findings from secondary care diabetes surveys in the UK have shown that there are significant variations in the facilities and resources available within hospitals to care for people with diabetes [9,10]. The aim of our study was to ascertain the structure of outpatient diabetes care in public hospitals in the Republic of Ireland and whether differences existed in services provided across hospitals.

## Methods

We conducted a cross-sectional observational study of the structure of adult outpatient diabetes care across all public hospitals in the Republic of Ireland. Institutions providing exclusively paediatric or maternity care were excluded, as were those where no diabetes outpatient clinics were provided. A letter of invitation to participate in the study was sent to all consultants involved in the provision of outpatient diabetes care in each hospital and copied to any specialist nursing staff for their information. The letter outlined the specific information required in relation to the provision of diabetes services locally and asked the consultant if he/she or another member of the diabetes team would be willing to participate in the survey. Once consent was given, a telephone interview utilising a survey instrument with open and closed questions was conducted in 2009. The survey instrument was developed with input from health care professionals delivering outpatient diabetes care in Ireland and was informed by a similar survey instrument used in a UK setting [10]. Data relating to staffing levels, types of clinics, waiting times and current liaison between hospital and primary care were recorded. Perceived engagement in quality improvement initiatives was also assessed, including staff training and education, structured patient education, audit and feedback and patient reminder systems. Ethical approval was given for the study by the Clinical Research Ethics Committee of the Cork Teaching Hospitals.

Chi squared or Fisher’s exact tests for proportions were performed on selected variables to explore differences in the structure of diabetes services led by an endocrinologist-compared to those led by a general physician. For the purposes of analysis a number of variables were re-categorised. Waiting times for newly diagnosed type 2 patients was dichotomised as under and over six months, the likelihood of discharging patients back into GP care was dichotomised as rarely/never or sometimes/usually and the whole time equivalent (WTE) of clinical nurse specialists was dichotomised as less than or two or more WTE. Engagement in quality improvement activities was dichotomised as either “always/usually” or “sometimes/never/rarely”. Statistical significance was accepted at the 5% level (p < 0.05).

## Results

At the time of data acquisition, 36 public hospitals were providing outpatient diabetes care to adults in the Republic of Ireland. Of these, individuals from 35 participated in the telephone interview. They comprised endocrinologists (n = 15), diabetes nurse specialists (n = 12), general physicians (n = 7) and one diabetes data manager.

Diabetes services were led by an endocrinologist in 21 hospitals (60%) and by a general physician in the remainder. A diabetes nurse specialist was present in 33 hospitals (94%). Hospitals with an endocrinologist were more likely to have two or more WTE specialist nurses than those with no endocrinologist (55% V 8%, p = 0.009). The WTE number of endocrinologists and clinical nurse specialists per 100,000 population were 0.72 and 1.2 respectively.

Thirty four hospitals provided data on access to support services. Twenty two hospitals (65%) reported having dedicated podiatry and 25 (74%) had dedicated dietetics for their outpatient diabetes care services. Retinal screening was available in six hospitals (18%). The remaining 82% either routinely referred all patients to an ophthalmologist for retinal screening (n = 15, 44%) or only did so if direct fundoscopy in the diabetes clinic was abnormal (n = 14, 41%). Access to support services in outpatient diabetes care by hospitals with and without endocrinologists is shown in Table 1.

**Table 1:** Access to support services in outpatient diabetes care by hospitals with and without endocrinologists

**Support service**	**With endocrinologist**	**No endocrinologist**	**All hospitals**	**p value**
	**(n = 21)**	**(n = 13)**	**(n = 34)**	
	**% (n)**	**% (n)**	**% (n)**	
Podiatrist	76 (16)	46 (6)	65 (22)	.14
Dietician	86 (18)	54 (7)	74 (25)	.06
Retinal screener	24 (5)	8 (1)	18 (6)	.37
Ophthalmologist	43 (9)	21 (3)	35 (12)	.28

Overall, 23 hospitals (66%) reported holding subspecialty diabetes clinics and this was more likely where an endocrinologist was in post (78% v 22%, p = 0.002). The most common clinics were diabetic foot clinics (44%, n = 10), young adult clinics (44%, n = 10), new patient clinics (39%, n = 9) and antenatal clinics (39%, n = 9). Most hospitals (86%, n = 30) reported delivering diabetes care in a dedicated general diabetes clinic. Fourteen percent (n = 5) reported seeing patients with diabetes in a general medical clinic setting only.

Thirty four hospitals provided information on waiting times for newly diagnosed type 2 patients (Table 2). Average waiting times exceeded six months according to 10 of 33 interviewees. Hospitals with an endocrinologist were more likely to have a wait greater than six months compared to those with a general physician (47% V 7%, p = 0.013).

**Table 2:** Waiting times for newly diagnosed type 2 patients by hospitals with and without endocrinologists

	**With endocrinologist**	**No endocrinologist**	**All hospitals**
	**(n = 21)**	**(n = 13)**	**n = 34**
	**% (n)**	**% (n)**	**% (n)**
< 3 months	20 (4)	71 (10)	41 (14)
3–6 months	30 (6)	21 (3)	26 (9)
7–12 months	35 (7)	0 (0)	21 (7)
12+ months	10 (2)	7 (1)	9 (3)
Other*	5 (1)	0 (0)	3 (1)

*One hospital reported two different waiting times (up to 3 months or up to 24 months) depending on the consultant that the patient has been referred to.

Stable patients (defined as those achieving treatment targets for glycaemic control, blood pressure and lipid profiles) were recalled to the diabetes outpatients routinely in 32 hospitals (91%). Similar recall times were reported by hospitals with and without endocrinologists with over three quarters of hospitals reporting recalling patients within 12 months.

Thirty five hospitals provided information on discharging patients back to GP care. In 20 centres (57%), patients were “rarely or never” discharged to primary care, while in 15 centres (43%) they were “sometimes or often” discharged. Hospitals with an endocrinologist were more likely to report “rarely or never” discharging patients back to primary care than those with a general physician (76 v 29%, p = 0.00**5)**.

All hospitals reported using formal clinical practice guidelines and 33 (94%) had specific treatment targets for glycaemic control, blood pressure and lipid profiles. Thirty-five hospitals provided information on involvement in quality improvement initiatives including staff and patient education and audit and feedback. Engagement in quality improvement initiatives by hospitals with and without an endocrinologist in post are shown in Table 3.

**Table 3:** Engagement in quality improvement initiatives by hospitals with and without endocrinologists

**Type of quality improvement initiative**	**With endocrinologist**		**No endocrinologist**		**Total**		**p value**
	**n = 21**		**n = 14**		**n = 35**		
	**% (n)**		**% (n)**		**% (n)**		
	**Always/usually**	**Sometimes/rarely/never**	**Always/usually**	**Sometimes/rarely/never**	**Always/usually**	**Sometimes/rarely/never**	
Health care professional training/education	86 (18)	14 (3)	57 (8)	43 (6)	74 (26)	26 (9)	.11
Structured patient education	95 (20)	5 (1)	79 (11)	21 (3)	89 (31)	11 (4)	.28
Assist patients in setting and attaining self-management goals	100 (21)	0 (0)	93 (13)	7 (1)	97 (34)	3 (1)	.40
Use patient held records to facilitate correspondence with patients	67 (14)	33 (7)	86 (12)	14 (2)	74 (26)	26 (9)	.26
Carry out audit and feedback	52 (11)	48 (10)	21 (3)	79 (11)	40 (14)	60 (21)	.09
Use patient reminder systems for upcoming hospital appointments	52 (11)	48 (10)	57 (8)	43 (6)	54 (19)	46 (16)	1.0

## Discussion

These findings demonstrate significant differences in the structure and delivery of outpatient diabetes services led by an endocrinologist compared to a general physician. Given that responses were received from 97% of hospitals approached, the data appear to be a valid representation of the profile of diabetes care in Ireland.

Recent reports in Ireland and the UK recommend that there should be two consultant endocrinologist posts and 1.6 WTE diabetes specialist nurses per 100,000 [11,12]. Our survey indicates that there is a shortage in the number of endocrinologists and specialist nurses in post with over a third of hospitals having no endocrinologist in post and overall a WTE of 0.72 endocrinologists and 1.2 specialist nurses per 100,000 population. Similar shortfalls in specialist staff have been reported in the UK with an estimated WTE of 0.76-1.00 endocrinologists and 1.1-1.25 WTE clinical nurse specialists per 100,000 population being reported [9].

The observation that a higher proportion of endocrinologist- led services had two or more specialist diabetes nurses may simply reflect the size and workload of the hospital diabetes outpatient population. Similarly endocrinologist-led services were also more likely to have sub-specialty clinics, which may in turn result in more specialist nursing staff to support these clinics. However, we cannot say this for certain from this study as data on the numbers and types of patients attending the outpatient clinics was either unavailable or inconsistent across hospitals and was not included in the analysis.

The absence of diabetes dietetic support in one quarter and podiatry in one third of centres as well as the availability of retinal screening in just 18% of centres suggest that aspects of hospital-based outpatient care could be developed further. Although these services are often delivered outside of hospital in other countries, community- based dietetic, podiatry and retinal screening services in Ireland are not widely available [7]. Prompt access to appropriate diabetes care has been shown to improve outcomes [3] so the fact that patients have to wait longer than six months for assessment in almost a third of hospitals is cause for concern. The finding that more patients had to wait over six months in centres with an endocrinologist compared to a general physician may simply reflect a tendency to refer most patients to an endocrinologist rather than a general physician.

The finding that patients were less likely to be discharged back to primary care from an endocrinologist- led service may be due to the fact that such patients have more complicated diabetes than those attending a general physician or a reluctance on the part of endocrinologists to discharge patients back into general practice. Determining whether variations in age, diabetes duration or co-morbidity account for differences in rates of discharge to primary care would be helpful. Nonetheless, at a time when the delivery of timely diabetes care that is community based as far as possible is being encouraged, the observation that this is achieved more where services are led by a general physician rather than an endocrinologist warrants further study.

## Conclusions

Variations exist in the structure and provision of diabetes care in Irish hospitals where endocrinology-led services have more developed subspecialty structures and access to specialist allied health professionals. Nonetheless, waiting times are longer and discharge rates to primary care are lower than for non-specialty led services. Further studies to determine the extent to which case-mix variation accounts for these observations are warranted. Hospital-based outpatient care could be developed further to ensure equitable services are provided nationally. At a time when the delivery of diabetes services in primary care is being promoted, further research is warranted on the factors influencing the successful transition to primary care.

## Competing interests

The authors declare that they have no competing interests.

## Authors’ contributions

MOD, ADS, MOM, DS, CB and SD made substantial contributions to the study design. MOD, ADS and MOM carried out the data collection and initial analysis of the data. MOD and FF led the writing of the manuscript. All authors were involved in the interpretation of the findings and reading and approving the final manuscript.

## Pre-publication history

The pre-publication history for this paper can be accessed here:

http://www.biomedcentral.com/1472-6963/13/493/prepub
